# Epidemiology and treatment outcome of nasopharyngeal carcinoma in a low-incidence population – a DAHANCA analysis in Denmark 2000–2018

**DOI:** 10.2340/1651-226X.2024.40499

**Published:** 2024-11-04

**Authors:** Erik Schiess, Kristian H. Jensen, Morten H. Kristensen, Jørgen Johansen, Jesper G. Eriksen, Christian Maare, Maria Andersen, Mohammad Farhadi, Christian R. Hansen, Jens Overgaard, Lisa L. Hjalgrim, Giedrius Lelkaitis, Jeppe Friborg

**Affiliations:** a Department of Oncology, Rigshospitalet, Copenhagen, Denmark; b Department of Oncology, Aarhus University Hospital, Aarhus, Denmark; c Department of Experimental Clinical Oncology, Aarhus University Hospital, Aarhus, Denmark; d Department of Oncology, Odense University Hospital, Odense, Denmark; e Department of Oncology, University Hospital Herlev, Herlev, Denmark; f Department of Oncology, Aalborg University Hospital, Aalborg, Denmark; g Department of Oncology, Zealand University Hospital Naestved, Næstved, Denmark; h Department of Pediatrics and Adolescent Medicine, Rigshospitalet, Copenhagen, Denmark; i Department of Pathology, Rigshospitalet, Copenhagen, Denmark

**Keywords:** Nasopharyngeal carcinoma, radiotherapy, Epstein-Barr virus, DAHANCA

## Abstract

**Introduction:**

Nasopharyngeal carcinoma (NPC) is a rare disease and most studies have therefore been conducted in endemic areas. The aim of this study was to describe epidemiology and treatment outcomes of NPC in a population-based, non-endemic setting.

**Material and methods:**

Patients with NPC diagnosed in Denmark from 2000 to 2018 were identified in the Danish Head and Neck Cancer Study Group (DAHANCA) database. Clinical records were reviewed to obtain missing data and confirm outcome, histological subtypes, Epstein-Barr virus (EBV)-status, prognostic factors, and treatment.

**Results:**

NPC was identified in 394 patients corresponding to age-standardized incidence rates of 0.5 and 0.2 per 100,000 in men and women, respectively. The 5-year overall (OS) and disease-specific survival (DSS) were 56 and 66%. In multivariate analysis, stage, smoking, and histology affected both OS and DSS, as patients with undifferentiated carcinomas had superior outcomes. Tumor EBV-status was determined in 221 patients, of whom 160 (72%) tested positive. EBV-positivity was associated with an improved OS in univariate analysis, but not after adjustment for relevant clinical factors.

**Interpretation:**

NPC is a rare malignancy in Denmark, and three in four patients have EBV-associated tumors. Tumor histology, smoking status, and stage, but not EBV-status, had independent prognostic impact on survival.

## Introduction

Nasopharyngeal carcinoma (NPC) is a malignancy arising from the mucosal epithelium of the nasopharynx. The epidemiology is distinctive with high incidence rates in specific populations in Southeast Asia, North Africa and among Inuit, while rare in most other populations [1]. NPC is divided into three major types: keratinizing squamous cell carcinoma (SCC), nonkeratinizing carcinoma, and basaloid SCC. Nonkeratinizing carcinoma is divided into differentiated and undifferentiated subtypes. The undifferentiated type is associated with Epstein-Barr virus (EBV) and ubiquitous in NPC endemic regions [2, 3]. Epidemiological studies have indicated that genetic, ethnic, and environmental factors contribute to the development of NPC. The epidemiology and prognosis of NPC have been extensively studied in endemic regions [3, 4]. However, these studies may not be generalized to non-endemic areas due to differences in etiologic factors, the distribution of histologic subgroups, and clinical experience. Only few population-based studies on NPC outcomes have been published from European countries [5–8]. These studies are limited either by a small cohort size, a lack of disease-specific outcome data, and/or outdated treatment methods. A recent Nordic study, which included patients treated in 2005–2009, found that survival and disease-control rates were inferior to those reported from NPC endemic regions; however, the majority of patients were not treated with modern radiation techniques [7]. Therefore, to adequately characterize the treatment outcomes of NPC in a non-endemic population over time, we initiated a complete, nationwide analysis of patients diagnosed with NPC in Denmark from 2000 to 2018.

## Material and methods

### Patient characteristics

Patients diagnosed with biopsy-proven NPC living in Denmark from 2000 to 2018 were included in the study. Data were extracted from the Danish Head and Neck Cancer Study Group (DAHANCA) database [9]. The DAHANCA database contains prospectively registered epidemiological and clinical information on all patients diagnosed with head and neck cancer in Denmark, including tumor characteristics, risk factors, treatment, and recurrence data. Patients living in Greenland or the Faroe Islands at time of diagnosis were excluded, as follow-up in these regions is not registered routinely in the DAHANCA database.

Medical records were reviewed in cases of missing data. National pathology register data was used for verification of recurrences.

During 2000–2018 different editions of the Tumor, Node, Metastasis (TNM) staging system has been used (UICC 6^th^, 7^th^, and 8^th^ editions). In the present cohort, patients have been re-staged to the Union for International Cancer Control (UICC) 8^th^ edition when possible.

### Pathology classification

Pathology descriptions on all patients diagnosed with NPC from 2000 to 2018 were retrospectively reviewed in the Danish Pathology Database (Patobank) to verify the diagnosis, characterize the histological subtype, and identify possibly non-registered recurrences. In the analyses, tumors were divided into *undifferentiate*d, *others,* and *unknown*. The presence of EBV was analyzed either by Epstein-Barr encoding region (EBER) in situ hybridization (EBER-ISH) or immunohistochemistry using EBV latent membrane protein 1 (LMP1-IHC) [10]. If both methods were performed, the results of the ISH were used.

### Treatment and follow-up

All patients receiving radical radiotherapy were treated according to the principles of DAHANCA. The DAHANCA radiotherapy guidelines have been continuously updated, with the most recent version from 2020 [11]. In brief, radical radiotherapy entailed treating the gross tumor volume (GTV) and high-dose clinical target volume 1 (CTV1) to 66–68 Gy in 33–34 fractions of 2 Gy. Five fractions per week were used through 2002, and since 2003, a moderately accelerated schedule with six fractions/week has been standard.

In 2000, all head-neck cancer centers in Denmark used 2D treatment techniques, and Intensity-Modulated Radiotherapy (IMRT) was gradually introduced from 2001 and fully implemented by 2007. The elective high-risk clinical target volume 2 (CTV2), defined as areas close to the GTV at high risk for microscopic disease, was prescribed a minimum dose of 60 Gy. The elective low-risk clinical target volume 3 (CTV3), defined as volumes at lower risk, but still with significant risk of microscopic disease (uninvolved lymph node levels II, III, IV, V, retropharyngeal), was prescribed a minimum dose of 50 Gy (46–48 Gy with 2D treatment technique). Patients in good performance status were offered concurrent chemotherapy with weekly cisplatin 40 mg/m^2^ during the whole inclusion period, but only routinely from 2006 and onwards.

A few patients may also have received adjuvant chemotherapy, but it was not possible to separate these from patients who received concurrent chemotherapy alone. Induction chemotherapy was also used in a small number of patients. Nimorazole, a hypoxic radiosensitizer, was offered to all patients and administered at an oral dose of 1,200 mg/m^2^ (maximum 2,500 mg) before each fraction [12].

Radiotherapy in palliative doses were primarily delivered as either 20 Gy/4fx, 30 Gy/10fx, 52 Gy/13fx or 56 Gy/14fx, with the first two schedules delivered with 5 fx/week and the last two with 2 fx/week.

During treatment, patients underwent clinical evaluations weekly. After treatment, patients were evaluated clinically at 2 weeks, 8 weeks, and every 4 months during years 1–2 and biannually during years 3–5 until 2015 and every 6 months during years 1–2 and annually during years 3–5 after 2014 [13]. Clinical follow-up evaluations included a physical examination and endoscopic pharyngo-laryngoscopy. Imaging was performed on clinical suspicion of recurrence and no guidelines for routine use were available during the study period.

### Statistical analysis

The crude rate of NPC was calculated using the annual January 1st Danish population (2000–2018) provided by Statistics Denmark [14]. Direct age-standardized incidence rates were calculated using the WHO standard population [15]. Applied endpoints were overall survival (OS) and disease-specific survival (DSS). OS was calculated from date of first admission to the oncology center and until date of death of any cause or until the date of last follow-up (Dec 31, 2023), whichever occurred first. DSS was calculated from date of first contact with the oncology center until date of death in patients with a recurrence or Dec 31, 2023, whichever occurred first, while patients dying without a recurrence were censored. Patients receiving primary palliative treatment were believed to have died of the cancer. Time-to-event analyses were calculated using the Kaplan–Meier method. The K-M method was chosen over a competing risk analysis in order to provide estimates that were easier to compare across populations. Cox-regression was applied in univariate and multivariate analyses, the latter adjusted for gender, age, stage (I–II, III, Iva, Ivb, unknown), histology (undifferentiated, others, and unknown), smoking status (never, former, and current), and primary treatment (radical radiotherapy, radical chemo-radiotherapy, and palliative treatment, the latter including palliative radiotherapy, palliative chemotherapy, and no treatment). The independent variables included in the multivariate analysis were selected a priori. A hazard ratio (HR) > 1 indicates a higher hazard rate of death in the specific subgroup compared to the reference group. The proportional hazards assumption was tested visually and by the evaluation of Schoenfeld residuals.

To illustrate temporal changes and for comparison, the cohort was divided into two periods 2000–2007 and 2008–2018. These periods were chosen as they roughly reflect before and after implementation of IMRT and concurrent chemotherapy.

Statistical survival analyses were performed using the statistical software R (2022-04-22, Vigorous Calisthenics) and R Studio (version 2021.09.0+351).

## Results

### The study cohort

The study identified 394 patients diagnosed in Denmark from 2000 to 2018 (Table 1 and Supplementary Figure 1). The male-to-female ratio was 2.6:1 and the median age at diagnosis was comparable in men and women (56 years). The majority of patients were diagnosed with undifferentiated carcinoma (292, 74%), followed by differentiated non-keratinizing SCC (34, 9%), non-keratinizing SCC NOS (16, 4%), keratinizing SCC (9, 2%), and basaloid SCC (2, 0.5%). In 41 (10%) patients the histology was unknown. The majority of patients were diagnosed in locally advanced stages (stage III–IVa, 63%), and lymph node involvement was observed in 73%. Primary metastatic disease was found in 29 (7%) patients. A total of 334 patients were planned for radical radiotherapy of which 331 had loco-regional disease.

**Table 1:** Characteristics of the study cohort.

	All patients (*N* = 394)	
**Gender**		
Men	285	72.3
Women	109	27.7
**T-classification**		
T1	168	42.6
T2	86	21.8
T3	45	11.4
T4	87	22.1
Unknown	8	2.0
**N-classification**		
N0	104	26.4
N1	97	24.6
N2	117	29.7
N3	74	18.8
Unknown	2	0.5
**M-classification**		
M0	365	92.6
M1	29	7.4
**Stage**		
I	43	10.9
II	74	18.8
III	123	31.2
IVa	124	31.5
IVb	29	7.4
Unknown	1	0.3
**Histology**		
Undifferentiated	292	74.1
Differentiated		
Differentiated non-keratinised	34	8.6
Keratinised	9	2.3
Basaloid	2	0.5
Non-keratinised (not classified)	16	4.1
Unknown	41	10.4
**Smoking**		
Never	112	28.4
Former	153	38.8
Current	117	29.7
Missing	12	3.0
**Primary treatment**		
Radical RT	111	28.2
Radical CRT	223	56.6
Palliative RT	40	10.2
Palliative CT	4	1.0
No treatment	16	4.1

RT: radiotherapy; CRT: chemo-radiotherapy; CT: chemotherapy.

The overall crude incidence rates for males and females were 0.6 and 0.2 pr. 100,000 years, respectively. The corresponding age-standardized rates were 0.5 per 100,000 years for males and 0.2 per 100,000 years for females. There was no clear indication of temporal changes in incidence rates over the period (Supplementary Figure 2).

Concurrent chemotherapy was administered to 223 patients (57%) (Table 1), with 17 patients (4%) also receiving induction chemotherapy in addition to the concurrent chemoradiation.

### Overall survival

The mean follow-up time was 11.3 years for patients still alive at last follow-up, and 3.9 years for patients who had died. The 5-year OS was 56%. A non-significant (*p* = 0.2) improvement in OS was observed between 2000–2007 (5-year OS: 51% CI: 43–59%) and 2008–2018 (5-year OS: 58% CI: 52–64%).

**Figure 1:**
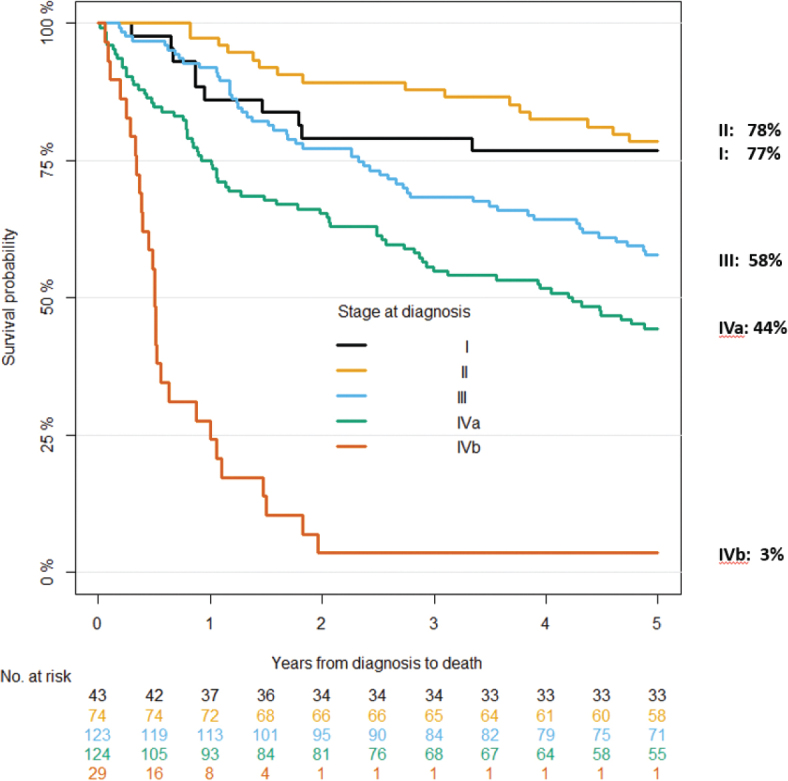
Overall survival of nasopharyngeal carcinoma patients in Denmark 2000–2018 according to stage (*n* = 394).

OS according to stages clearly differed with decreasing 5-year survival in higher stages, except between stages I and II (I: 77%, II:78%, III:56%, IVa:45%, IVb:3%, Figure 1).

A significant association was found between OS and age, smoking, histology, stage, and primary treatment in univariate analysis (Table 2). The effects of these variables were maintained in the multivariate model except for concurrent chemotherapy that was no longer significant.

**Table 2:** Factors associated with overall survival and disease-specific survival of NPC patients in Denmark 2000–2018.

Analysis	*n*	Overall survival						Disease-specific survival					
		Univariate			Multivariate			Univariate			Multivariate		
		HR	95% CI	*p*	HR	95% CI	*p*	HR	95% CI	*p*	HR	95% CI	*p*
**Variable**													
**Age**	394	1.04	1.0; 1.1	<0.001	1.03	1.0; 1.1	<0.001	1.02	1.01; 1.04	0.004	1.02	1.01; 1.03	0.010
**Sex**													
Male	285	1	ref										
Female	109	0.94	0.7; 1.3	0.69	1.10	0.8; 1.5	0.52	0.88	0.6; 1.3	0.48	1.03	0.7; 1.5	0.88
**Smoking status**													
Never-smoker	112	1	ref										
Former-smoker	153	2.36	1.6; 3.4	<0.001	1.30	0.9; 1.9	0.20	2.43	1.5; 3.9	<0.001	1.30	0.8; 2.2	0.31
Current-smoker	117	3.60	2.5; 5.2	<0.001	2.83	1.9; 4.3	<0.001	3.34	2.1; 5.4	<0.001	2.51	1.5; 4.1	<0.001
Unknown	12												
**Histological type**													
Undifferentiated	292	1	ref										
Others	61	2.07	1.5; 2.9	<0.001	1.64	1.2; 2.3	0.005	2.0	1.3; 3.0	0.001	1.72	1.1; 2.7	0.015
Unknown	41	4.27	3.0; 6.2	<0.001	3.09	2.1; 4.7	<0.001	3.4	2.1; 5.4	<0.001	2.48	1.5; 4.2	<0.001
**Stage**													
I+II	117	1	ref										
III	123	1.49	1.0; 2.1	0.031	1.62	1.1; 2.4	0.014	1.90	1.1; 2.9	0.010	1.91	1.1; 3.2	0.013
IVa	124	2.29	1.6; 3.2	<0.001	2.27	1.6; 3.3	<0.001	2.72	1.7; 4.4	<0.001	2.31	1.4; 3.8	0.001
IVb	29	13.49	8.2; 22.1	<0.001	12.68	6.7; 23.9	<0.001	26.35	14.8; 46.8	<0.001	15.67	7.5; 32.6	<0.001
Unknown	1												
**Primary treatment**													
Radical RT	111	1	ref										
Radical CRT	223	0.58	0.4; 0.8	<0.001	0.83	0.6; 1.1	0.26	0.72	0.5; 1.1	0.11	0.91	0.6; 1.4	0.66
Palliative treatment*	60	4.42	3.1; 6.3	<0.001	1.59	1.0; 2.5	0.043	6.95	4.5; 10.7	<0.001	3.01	1.8; 5.5	<0.001

* Palliative treatment includes palliative RT, palliative chemotherapy, and no treatment.

HR for *n* < 5% of total sample not shown. HR for age per 1-year increase.

Multivariate models include variables age, sex, stage, histology, smoking, and chemotherapy.

NPC: nasopharyngeal carcinoma; RT: radiotherapy; CRT: chemo-radiotherapy; HR: hazard ratio.

Restricting the cohort to only include patients treated with curative intent, defined by a planned dose of 66–68 Gy and no distant metastases at diagnosis (*n* = 331), the corresponding 5-year OS was 63% (59–69%), and in 2000–2007 and 2008–2018, 60% (51–68%) and 66% (60–72%), respectively.

### Disease-specific survival

**Figure 2:**
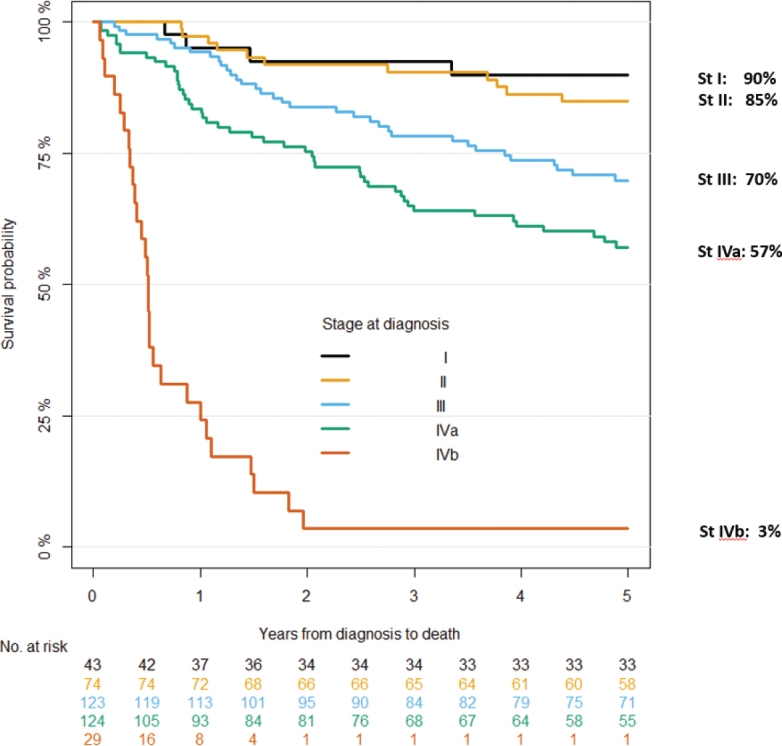
Disease-specific survival of nasopharyngeal carcinoma patients in Denmark 2000–2018 according to stage (*n* = 394).

The 5-year DSS for the full cohort was 66% CI [61–71%]. There was a numerical improvement in DSS between 2000–2007 (5 year DSS: 61% CI: 53–70%) and 2008–2018 (5-year DSS: 69% CI: 63–75%), but the difference was not statistically significant (*p* = 0.2). DSS according to stages clearly differed with decreasing 5-year survival in higher stages (I: 90%, II:85%, III:70%, IVa:57%, IVb:3%, Figure 2).

In univariate analysis, increasing age, smoking, histology, stage, and palliative treatment were significantly associated with a higher risk of dying from NPC (Table 2). In multivariate analysis, the effects of these variables were maintained. Restricting the cohort to include only patients treated with curative intent (*n* = 331), the corresponding 5-year DSS was 75% (95% CI:71–80%), and in 2000–2007 and 2008–2018, 71% (95% CI:63–80%) and 78% (95% CI:72–84%), respectively.

## EBV-status

**Figure 3:**
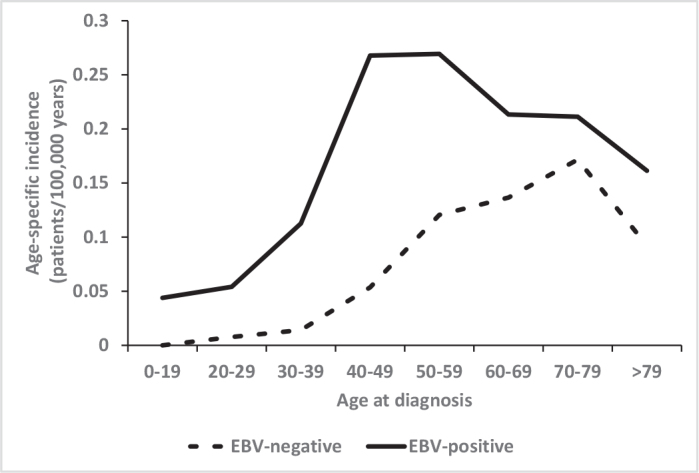
Age-specific incidence of nasopharyngeal carcinoma (NPC) 2000–2018 according to known Epstein-Barr virus (EBV)-association (*n* = 221).

EBV status in tumor was determined in 221 patients, with an overall EBV-positivity of 72% (160/221, Table 3). EBER in-situ hybridization was used in 167 (76%) of EBV-analyses, LMP1 immunohistochemistry in 25 (11%) and in 29 (13%) the method of analysis was unknown. EBV-testing became more prevalent over time with 30% of patients tested over 2000–2007 and 71% over 2008–2018. A number of differences between EBV-positive and -negative NPC were observed. EBV-positive patients were on average 10 years younger than EBV-negative patients (mean age 50.2 and 60.4 years), illustrated by the earlier plateau in age-specific incidence rates (Figure 3). Most EBV-positive NPCs were undifferentiated carcinomas (95%), but 21% of all undifferentiated carcinomas were EBV-negative (Table 3). EBV-positive NPC presented with lower T-classifications, but there was no significant difference in the distribution of N-classification or stage between EBV-positive and -negative (Table 3).

**Table 3:** Characteristics of EBV-tested patients with nasopharyngeal carcinoma (*n*=221).

	EBV negative	EBV positive	All EBV tested patients
**All patients**	61 (27.6%)	160 (72.4%)	221 (100%)
**Gender**			
Men	43 (70.5%)	116 (72.5%)	159 (71.9%)
Women	18 (29.5%)	44 (27.5%)	62 (28.1%)
**Stage**			
I	6 (9.8%)	18 (11.3%)	24 (10.9%)
II	8 (13.1%)	37 (23.1%)	45 (20.4%)
III	18 (29.5%)	52 (32.5%)	70 (31.7%)
IVa	25 (41.0%)	46 (28.8%)	71 (32.1%)
IVb	4 (6.6%)	7 (4.4%)	11 (5.0%)
**Histology**			
*Undifferentiated*	41 (67.2%)	151 (94.4%)	192 (86.9%)
*Differentiated:*			
Differentiated non-keratinised	13 (21.3%)	5 (3.1%)	18 (8.1%)
Keratinised	1 (1.6%)	0 (0%)	1 (0.5%)
Basaloid	1 (1.6%)	0 (0%)	1 (0.5%)
*Non-keratinised (not classified)*	3 (4.9%)	3 (1.9%)	6 (2.7%)
*Unknown*	2 (3.3%)	1 (0.6%)	3 (1.4%)
**Smoking**			
Never	13 (21.3%)	65 (40.6%)	78 (35.3%)
Former	30 (49.2%)	56 (35.0%)	86 (38.9%)
Current	18 (29.5%)	36 (22.5%)	54 (24.4%)
Unknown	0 (0%)	3 (1.9%)	3 (1.4%)
**Primary treatment**			
Radical RT	14 (23.0%)	31 (19.4%)	45 (20.4%)
Radical CRT	39 (63.9%)	117 (73.1%)	156 (70.6%)
Palliative RT	5 (8.2%)	8 (5.0%)	13 (5.9%)
Palliative CT	1 (1.6%)	1 (0.6%)	2 (0.9%)
No treatment	2 (3.3%)	3 (1.9%)	5 (2.3%)

RT (radiotherapy), CRT (chemo-radiotherapy), CT (chemotherapy)

OS was higher in EBV-positive NPC compared to EBV-negative with a 5-year OS of 74% (95% CI: 68–81%) and 57% (95% CI: 45–70%), *p* < 0.001. This was confirmed in a univariate Cox-regression analysis where EBV-positivity was found to be a significant positive prognostic factor with a HR of 0.47 (95% CI: 0.3;0.7, *p* < 0.001) (Supplementary Table 1). After adjustment for age, gender, smoking, stage, and primary treatment, the effect of EBV disappeared with a HR of 0.76 (95% CI: 0.5;1.2, *p* = 0.25).

Likewise, EBV-status was associated with DSS in univariate, but not multivariate analysis (Supplementary Table 1).

### Primary metastatic

Twenty-nine patients presented with metastatic disease at the time of diagnosis. The mean and median survival times were 9 and 6 months, respectively. There was no significant influence of EBV-status on OS in patients with metastatic disease. One patient with primary metastatic NPC involving the mediastinum and pulmonary hila was alive at the end of follow-up, more than 3 years after diagnosis.

### Juvenile NPC

A total of 17 patients (4.3%) were younger than 20 years of age at diagnosis. The histology was undifferentiated carcinoma in 88%, differentiated non-keratinized SCC in one patient (6%), and unknown in one (6%). The 11 patients tested for EBV in tumor, were all positive (100%). The 5-year OS and DSS was 88%. Two patients died of cancer, one with primary metastatic disease (stage IVb) and one with metastatic recurrence 14 months after diagnosis.

## Discussion

The present study is a large, complete population-based survey of NPC epidemiology and outcome in a low-incidence population. The results of this study confirm the rarity of the NPC in Denmark and indicate improvements in treatment outcome over the past 20 years.

With crude and age-standardized incidence rates well below 1 per 100,000, the frequency of NPC in Denmark is very low and is comparable to rates reported from the other Scandinavian and Northern European countries [3]. This contrasts with the frequencies reported in Chinese, Southeast Asian, North African, and Inuit populations, with incidence rates up to 20 per 100,000. Compared to Greenland, which is part of the Danish kingdom, NPC is 40 times more frequent among Inuit [16]. Although patients from Greenland are treated in Denmark, the present study did not include patients living outside Denmark at the time of diagnosis.

In endemic populations, a declining incidence of NPC has been reported, partly attributed to changes in lifestyle and economic development [3]. In a recent national cancer registry study covering 1980–2014, a non-significant decline of 3% annually was observed [8]. Differences in etiological factors may explain the different temporal trends in NPC incidence rates observed in high- and low-incidence regions. NPC is believed to be the result of environmental factors acting on genetically susceptible individuals. The weak genetic disposition for NPC in low-incidence areas combined with the lack of substantial socio-economic changes in Denmark over the last 50 years may account for the very modest change in incidence rates.

The median age at diagnosis of NPC patients in Denmark is 56 years, which is 5–10 years less than HPV-negative SCCs of the oropharynx [17]. This is consistent with the knowledge from NPC high-incidence populations that traditional carcinogenic lifestyle factors like smoking and alcohol consumption exert a limited influence on the risk of NPC [18], though tobacco consumption affects survival as demonstrated in this series.

While virtually all NPC in high-incidence regions are low-/undifferentiated carcinomas [3], the histological presentation is more varied in low-incidence populations. In Finland, Ruuskanen et al. found only 61% of NPC to be undifferentiated carcinomas, and in a North American population >50% of patients had differentiated carcinomas [19, 20]. Ruuskanen et al. reviewed all pathology slides, whereas this was not done by Vaughan et al., which may partly explain the discrepancy. In the current study, all pathology descriptions were reviewed, and three out of four carcinomas were found to be undifferentiated, supporting the fact that undifferentiated NPC constitute the majority of NPCs in low-incidence populations.

NPC is a challenging disease to treat because of the anatomical proximity to the skull base, brainstem, and optic structures, and radiotherapy is the primary treatment. Due to the rarity of NPC in Denmark, obtaining and maintaining adequate treatment experience may be challenging; however, national guidelines have helped to standardize treatment planning and delivery [11].

In the current study, the 5-year OS increased from 51 to 58% and DSS from 61 to 69% in 2000–2007 and 2008–2018 respectively. By contrast, the first Danish study on NPC, which included patients treated between 1963 and 1991, reported a 5-year OS of 43% and DSS of 50% [21]. In the combined Nordic study 2005–2009, the 5-year OS was 60.2% and DSS was 66.4% comparable to the 5-year OS of 60.5% 2010–2014 reported in a Danish registry study of all nasopharyngeal tumors [7, 8]. In Finland, Ruuskanen et al. [7] reported a 5-year OS and DSS of 63 and 66% in 2000–2009 in patients treated with curative intent. The corresponding 5-year OS and DSS for patients in this study treated with curative intent were 60 and 71% in 2000–2008, and 66 and 78% in 2009–2018. In a population-based cohort from Taiwan over 2002–2010 a 5-year OS of 65.2% for all patients regardless of treatment modality was observed [22].

All these survival rates are considerably lower than contemporary results from endemic areas; for example, in more than 8,000 patients treated with radical intent from 2009 to 2015 in Guangzhou with a 5-year OS above 85% [23]. But the challenges of comparing treatment results across populations are highlighted by the fact that the median age in the Chinese cohort was 45 years, 65% were never smokers, and more than 85% received chemo-radiation. However, we believe that contemporary treatment outcomes in Denmark are equal to those reported from comparable populations.

The addition of chemotherapy to radiotherapy has been associated with a survival benefit in the treatment of NPC, with cisplatin being the most commonly used drug [4, 24]. This study did not find a significant effect of concurrent chemotherapy on survival in a multivariate model, but the HR (0.87 OS) was comparable to the results from clinical trials [25]. This indicates an effect of chemotherapy in NPC; however, larger cohorts are needed to evaluate this. Another explanation for the lack of effect is the routine use of concurrent chemotherapy to stage I NPC in Denmark. This is not recommended in the ESMO clinical guidelines [24] and the likely small benefit in stage I patients may dilute the overall estimates.

Only few patients up to 2018 received induction chemotherapy, and a focused analysis of the effect of this was impossible in the current material. Recent studies have shown a significant survival benefit of induction chemotherapy followed by chemo-radiotherapy in selected patients with stage III/IV non-keratinized NPC [26]. This effect was primarily mediated through a reduction in the risk of distant metastases and will likely increase the survival of NPC patients in the future.

EBV is an established risk factor for NPC, especially the low-differentiated/undifferentiated types. While ubiquitous in NPC endemic populations, the prevalence of EBV in NPC from non-endemic populations is more varied. EBER in-situ hybridization is considered the gold standard of EBV detection in carcinomas [10]. An alternative method used earlier is immunohistochemistry using antibodies against EBV LMP1 which can be expressed on the surface of cells infected with EBV. The sensitivity of this analysis in NPC is lower than EBER in-situ hybridization, which may account for the inconsistent results in non-endemic populations, with EBV-positivity ranging from 0 to 83% [27]. However, two recent studies using EBER in-situ hybridization from Finland and the Czech Republic found 62 and 86% of all NPC EBV-positive, with 85% of the undifferentiated type EBV-positive in the Finnish cohort [19, 27]. These proportions are comparable to our results with an overall EBV-positivity of 74%, indicating that the majority of NPC in non-endemic regions are indeed EBV-associated. This also suggests that many NPC patients in non-endemic populations could benefit from the prognostic and surveillance progress brought by the analysis of free EBV-DNA in plasma [28, 29].

The clinical significance of EBV in NPC from non-endemic regions is debatable and EBV has been found to be a positive prognostic factor for survival in NPC in some studies [18, 30]. In this study, EBV-positivity was a strong positive prognostic factor in univariate, but not in a multivariate analysis incorporating relevant clinical factors, such as stage, age, and smoking. This is consistent with results from an American study [31] where the positive association of EBV disappeared after adjustment for relevant clinical factors. These results underline the importance of controlling for other clinical factors when analyzing the prognostic influence of EBV in non-endemic NPC.

The current study benefits from national prospectively collected data and validation through national registries. A weakness is the inconsistent frequency of EBV-analysis across the study period and the lack of a central reassessment of pathology slides for the histological classification, as classification was based solely on the original pathology description.

In conclusion, in this non-endemic region, three-fourths of Danish patients with NPC have EBV-positive tumors. Tumor histology, smoking, and disease stage were significantly associated with overall and DSS rates, and disease outcome comparable to other NPC low-incidence populations.

## Supplementary Material

Epidemiology and treatment outcome of nasopharyngeal carcinoma in a low-incidence population – a DAHANCA analysis in Denmark 2000–2018

## Data Availability

Data from the Danish Head and Neck Cancer Study Group (DAHANCA) database under the Danish Clinical Quality Program – National Clinical Registries (RKKP) was used for the study. Because of restrictions of data availability, data are not publicly available.
