# Supplementary material for: Epidemiology and treatment outcome of nasopharyngeal carcinoma in a low-incidence population – a DAHANCA analysis in Denmark 2000–2018
Source: Acta Oncol. 2024 Nov 4;63:40499. doi: 10.2340/1651-226X.2024.40499 (PMC11541798; doi:10.2340/1651-226X.2024.40499)
Supplement: Epidemiology and treatment outcome of nasopharyngeal carcinoma in a low-incidence population – a DAHANCA analysis in Denmark 2000–2018 [file AO-63-40499-s1.pdf]

Supplementary material has been published as submitted. It has not been copyedited, or typeset by Acta Oncologica

Supplementary data

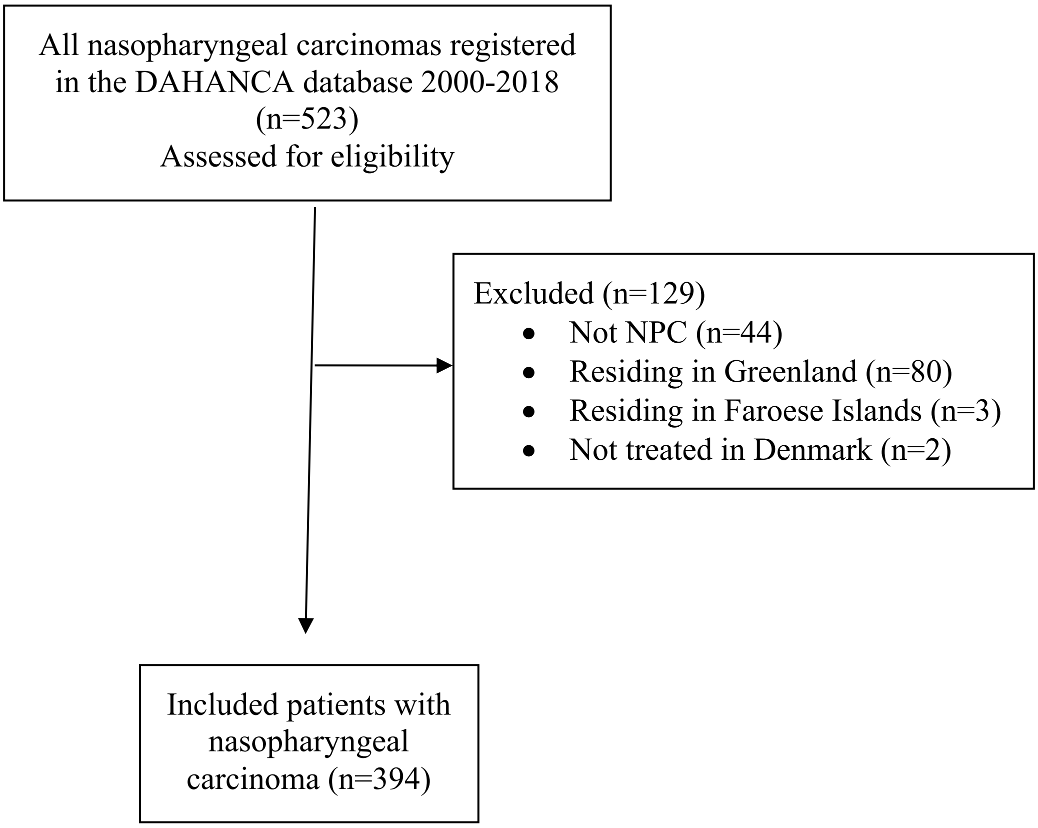

Figure 1: Diagram of included patients

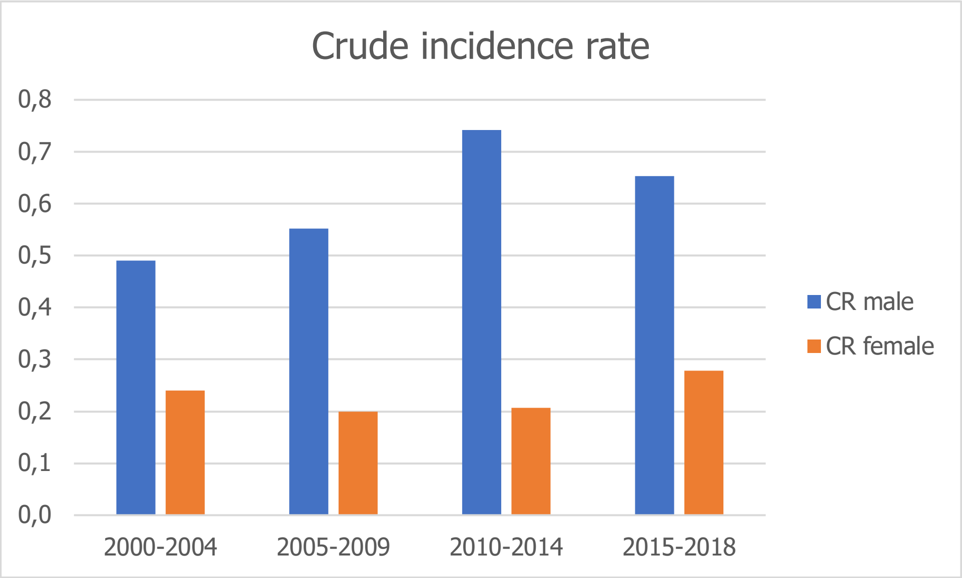

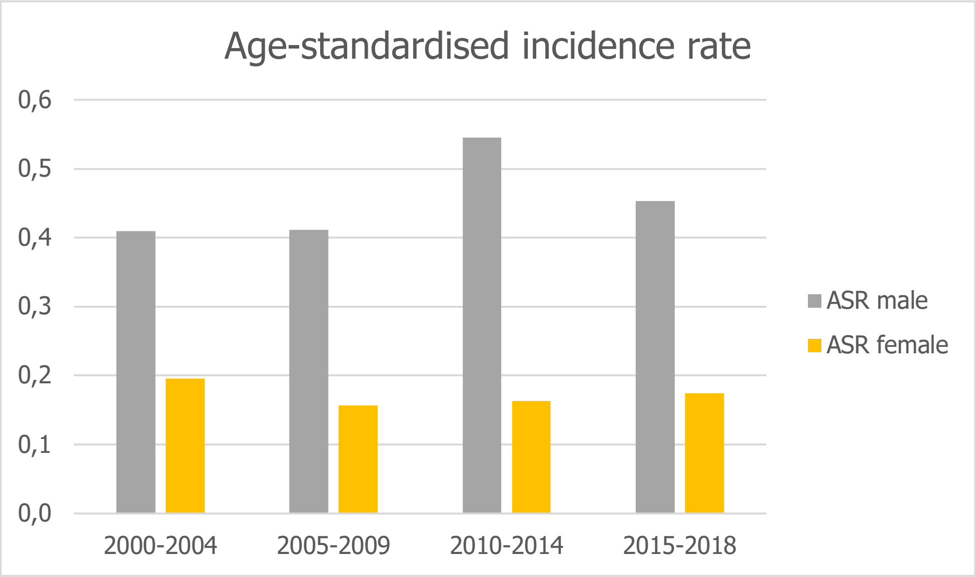

Figure 2: Crude rate and age-standardised incidence rate (patients/100,000 person years)

Table 1: Hazard ratio (HR) for overall survival and disease-specific survival according to EBV-status.

		Univariate analysis			Multivariate analysis ^		
	n	HR	CI	p-value	HR	CI	p-value
**Overall survival**							
EBV negative	61	1 (ref)			1 (ref)		
EBV positive	160	0.47	[0.3;0.7]	<0.001	0.76	[0.5;1.2]	0.25
**Disease-specific survival**							
EBV negative	61	1 (ref)					
EBV positive	160	0.57	[0.3;1.0]	0.033	0.97	[0.5;1.8]	0.92
^Adjusted for age, gender, smoking, stage and primary treatment.							
